# Supplementary material for: Knockdown of 15-bp Deletion-Type v-raf Murine Sarcoma Viral Oncogene Homolog B1 mRNA in Pancreatic Ductal Adenocarcinoma Cells Repressed Cell Growth In Vitro and Tumor Volume In Vivo
Source: Cancers (Basel). 2022 Jun 28;14(13):3162. doi: 10.3390/cancers14133162 (PMC9264874; doi:10.3390/cancers14133162)
Supplement: Supplementary file 1 [file cancers-14-03162-s001.zip › cancers-1731130-supplementary.pdf]

Supplementary Materials:

Supplementary Figures and Tables:

Figure S1. RNAi and off-target activities of each siRNA targeting BRAF in AsPC-1 cells determined by dual luciferase reporter assays.
Figure S2. RNAi and off-target activities of each siRNA targeting BRAF in OCUB-M cells determined by dual luciferase reporter assays.
Figure S3. Validation of microarray data by qRT-PCR.
Figure S4. Effects of unmodified and 2′-OMe-modified siBRAFs on the endogenous WT and Mut BRAF mRNA expression.
Figure S5. Detection of innate immune response by siRNA.
Figure S6. Differences in siRNA stabilities between unmodified and 2′-OMe-modified siRNAs.

Table S1. SiRNA sequences used for reporter assays, qRT-PCRs, Western Blots.
Table S2. Oligonucleotides sequences inserted into psiCHECK-1 for luciferase reporter assays.
Table S3. Primer sequences used for qRT-PCRs.

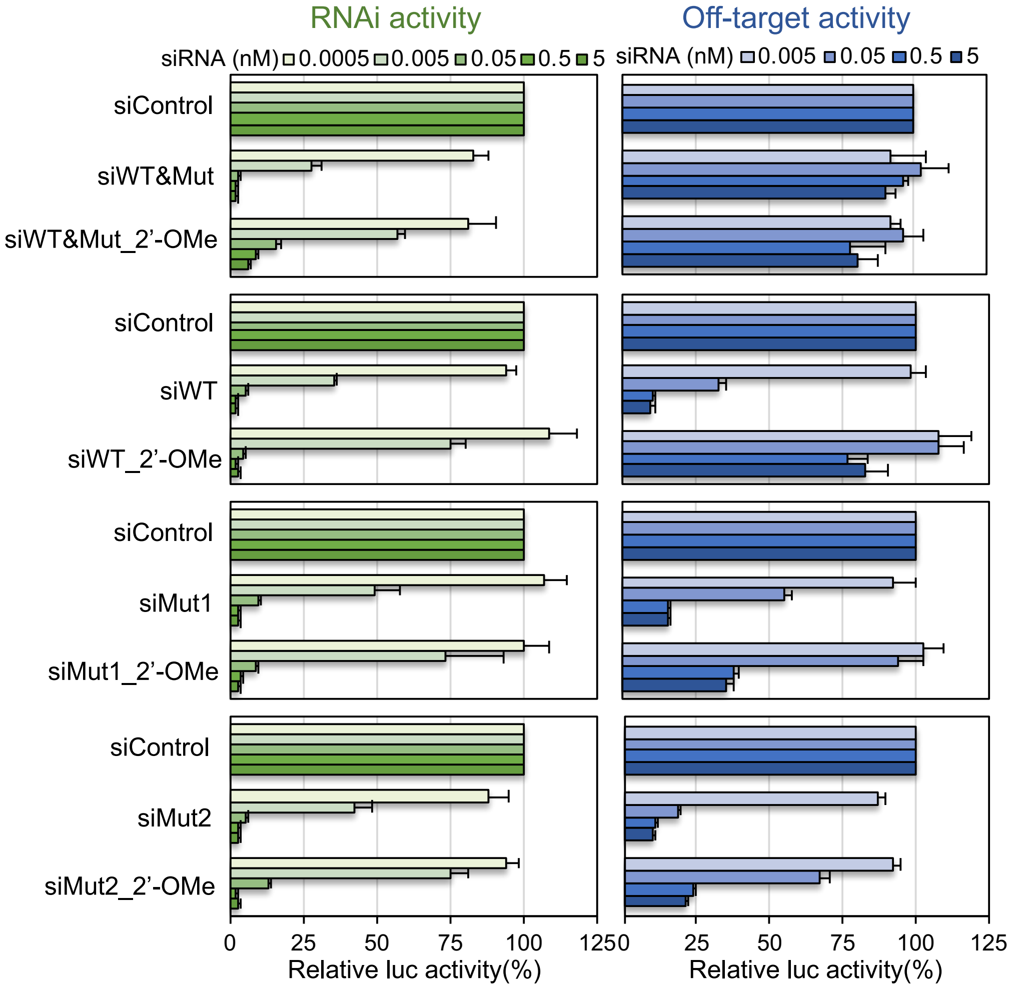

Figure S1. RNAi and off-target activities of each siRNA targeting BRAF in AsPC-1 cells determined by dual luciferase reporter assays. The CM (RNAi) or SM (off-target) reporter constructs were transfected with an siRNA along with the pGL3-Control firefly luciferase expression construct into AsPC-1 cells, and luciferase activity was measured 1 day after transfection. The RNAi and off-target activities were calculated as *Renilla* luciferase activity normalized to control firefly luciferase activity. RNAi (left, green bars) and off-target activities (right, blue bars) for five siRNA concentrations (0.0005, 0.005, 0.05, 0.5, and 5 nM). The horizontal bars indicate the relative luciferase activity levels.

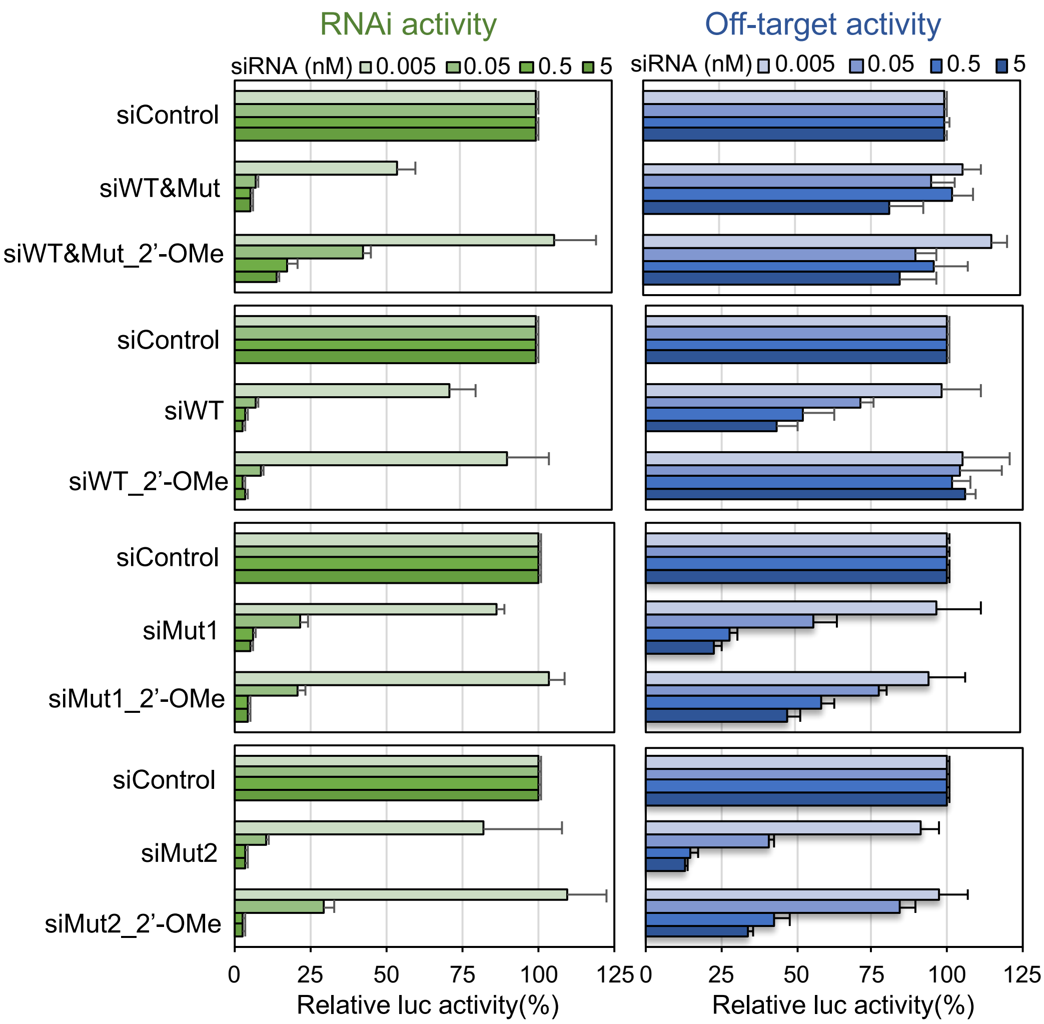

Figure S2. RNAi and off-target activities of each siRNA targeting BRAF in OCUB-M cells determined by dual luciferase reporter assays. The CM (RNAi) or SM (off-target) reporter constructs were transfected with an siRNA along with the pGL3-Control firefly luciferase expression construct into OCUB-M cells, and luciferase activity was measured 1 day after transfection. The RNAi and off-target activities were calculated as *Renilla* luciferase activity normalized to control firefly luciferase activity. RNAi (left, green bars) and off-target activities (right, blue bars) for four siRNA concentrations (0.005, 0.05, 0.5, and 5 nM). The horizontal bars indicate the relative luciferase activity levels.

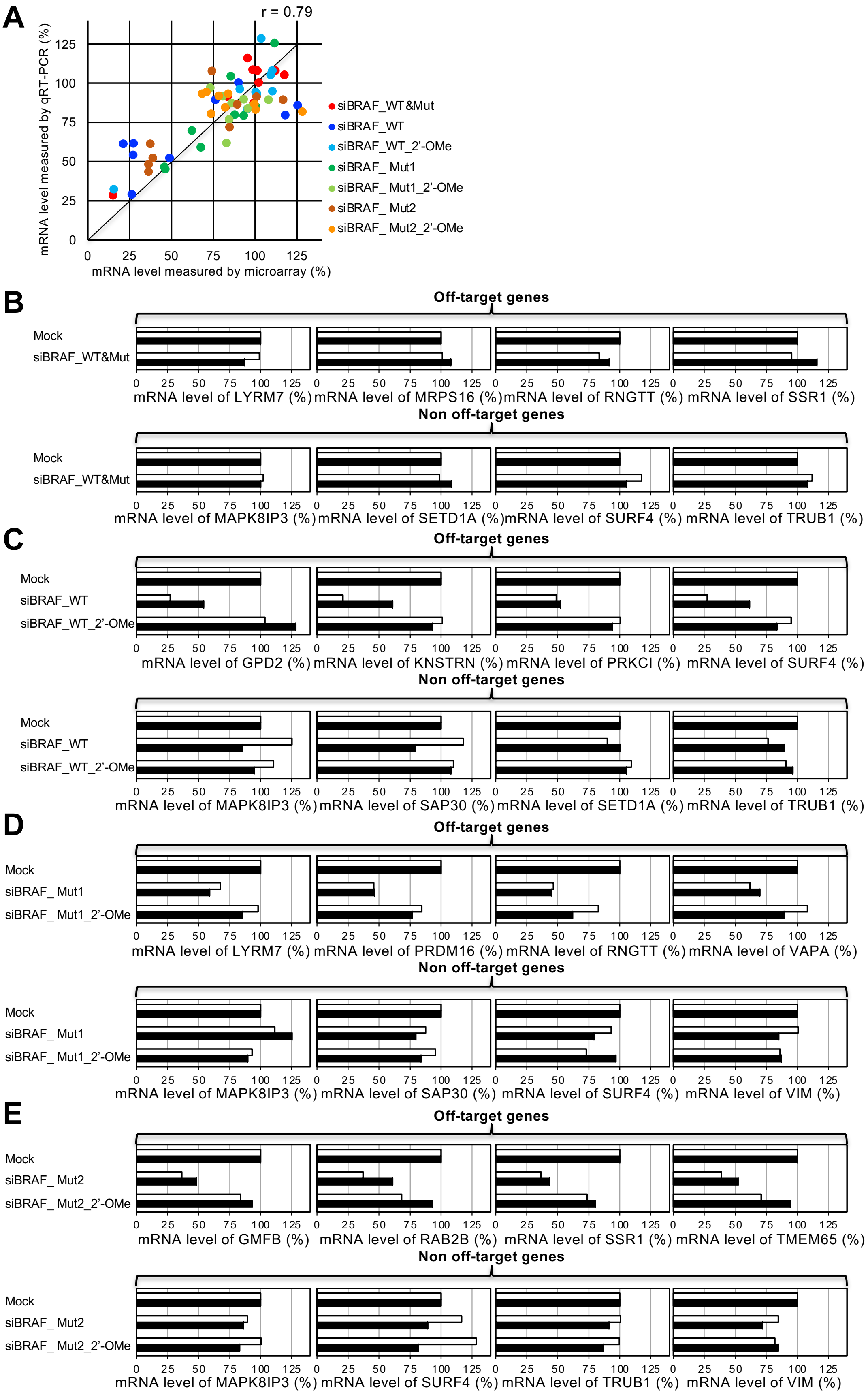
A
r = 0.79
mRNA level measured by qRT-PCR (%)
mRNA level measured by microarray (%)
siBRAF_WT&Mut
siBRAF_WT
siBRAF_WT_2'-OMe
siBRAF_ Mut1
siBRAF_ Mut1_2'-OMe
siBRAF_ Mut2
siBRAF_ Mut2_2'-OMe
B
Off-target genes
Mock
siBRAF_WT&Mut
mRNA level of LYRM7 (%)
mRNA level of MRPS16 (%)
mRNA level of RNGTT (%)
mRNA level of SSR1 (%)
Non off-target genes
mRNA level of MAPK8IP3 (%)
mRNA level of SETD1A (%)
mRNA level of SURF4 (%)
mRNA level of TRUB1 (%)
C
Off-target genes
Mock
siBRAF_WT
siBRAF_WT_2'-OMe
mRNA level of GPD2 (%)
mRNA level of KNSTRN (%)
mRNA level of PRKCI (%)
mRNA level of SURF4 (%)
Non off-target genes
mRNA level of MAPK8IP3 (%)
mRNA level of SAP30 (%)
mRNA level of SETD1A (%)
mRNA level of TRUB1 (%)
D
Off-target genes
Mock
siBRAF_ Mut1
siBRAF_ Mut1_2'-OMe
mRNA level of LYRM7 (%)
mRNA level of PRDM16 (%)
mRNA level of RNGTT (%)
mRNA level of VAPA (%)
Non off-target genes
mRNA level of MAPK8IP3 (%)
mRNA level of SAP30 (%)
mRNA level of SURF4 (%)
mRNA level of VIM (%)
E
Off-target genes
Mock
siBRAF_ Mut2
siBRAF_ Mut2_2'-OMe
mRNA level of GMFB (%)
mRNA level of RAB2B (%)
mRNA level of SSR1 (%)
mRNA level of TMEM65 (%)
Non off-target genes
mRNA level of MAPK8IP3 (%)
mRNA level of SURF4 (%)
mRNA level of TRUB1 (%)
mRNA level of VIM (%)

Figure S3. Validation of microarray data by qRT-PCR. (A) Four off-target genes with SM sequences with each of siBRAF_WT&Mut, siBRAF_WT, siBRAF_Mut1, or siBRAF_Mut2 in their 3'UTRs were arbitrarily chosen, and four genes without SM sequences were also chosen as negative controls. Changes in expression levels of their mRNAs in siBRAF-treated HeLa cells, compared with mock transfection, were examined by qRT-PCR and microarray (B-F; see also Figure 4). Red dots indicate data obtained from the cells transfected with unmodified siBRAF_WT&Mut. Blue or light blue dots, data from the cells transfected tih unmodified or 2′-OMe-modified siBRAF_WTs. Green or light green dots, siBRAF_Mut1s. Brown or orange dots, siBRAF_Mut2s. The correlation coefficient was estimated at 0.79. Comparison at the level of individual gene is shown in (B-E). (B) Transcripts with or without 3′ UTRs possessing SM sequences of siBRAF_WT&Mut; *LYR motif containing 7* (LYRM7), *mitochondrial ribosomal protein S16* (MRPS16), *RNA guanylyltransferase and 5'-phosphatase* (RNGTT), *signal sequence receptor subunit 1* (SSR1) have siBRAF_WT&Mut SM sequences (Off-target genes), *mitogen-activated protein kinase 8 interacting protein 3* (MAPK8IP3), *SET domain containing 1A, histone lysine methyltransferase* (SETD1A), *surfeit 4* (SURF4), and *TruB pseudouridine synthase family member 1* (TRUB1) do not have siBRAF_WT&Mut SM sequences (Not off-target genes). (C) Transcripts with or without SM sequences of siBRAF_WT; *glycerol-3-phosphate dehydrogenase 2* (GPD2), *kinetochore localized astrin (SPAG5) binding protein* (KNSTRN), *protein kinase C iota* (PRKCI), SURF4 are off-target genes, MAPK8IP3, *Sin3A associated protein 30* (SAP30), SETD1A, and TRUB1 are not off-target genes. (D) Transcripts with or without seed-match sequence of siBRAF_Mut1; LYRM7, *PR/SET domain 16* (PRDM16), RNGTT, *VAMP associated protein A* (VAPA) are off-target genes, MAPK8IP3, SAP30, SURF4, and *vimentin* (VIM) are not off-target genes. (E) Transcripts with or without SM sequences of siBRAF_Mut2; *glia maturation factor beta* (GMFB), *RAB2B, member RAS oncogene family* (RAB2B), SSR1, *transmembrane protein 65* (TMEM65) are off-target genes, MAPK8IP3, SURF4, TRUB1, and VIM. White bars indicate the relative signal intensity (Microarray data). Black bars indicate the relative expression level (qRT-PCR data) are not off-target genes.

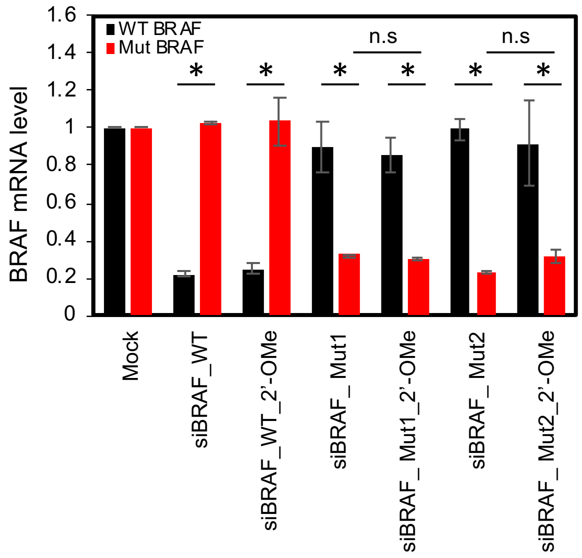

Figure S4. Effects of unmodified and 2′-OMe-modified siBRAFs on the endogenous WT and Mut BRAF mRNA expression. WT and Mut BRAF mRNA levels quantified by qRT-PCR using total RNA purified from BxPC-3 cells transfected with each siRNA according to the same procedure shown in Figure 2B. Specific primers were used to measure WT and Mut BRAF mRNA levels separately (Figure 2C). The unmodified and 2′-OMe-modified siBRAF_WTs significantly suppressed WT BRAF mRNA; both of unmodified siBRAF_Mut1 and 2′-OMe-modified siBRAF_Mut1_2′-OMe significantly suppressed Mut BRAF mRNA level. Furthermore, there was almost no difference in the Mut BRAF mRNA levels by the transfection of siBRAF_Mut2 or siBRAF_Mut2_2′-OMe. The *p*-values for comparisons of WT and Mut BRAF mRNA levels were determined using Student's *t*-test ($*p < 0.05$). Their mRNA levels were normalized to that in mock-transfected control cells. n.s., not significant.

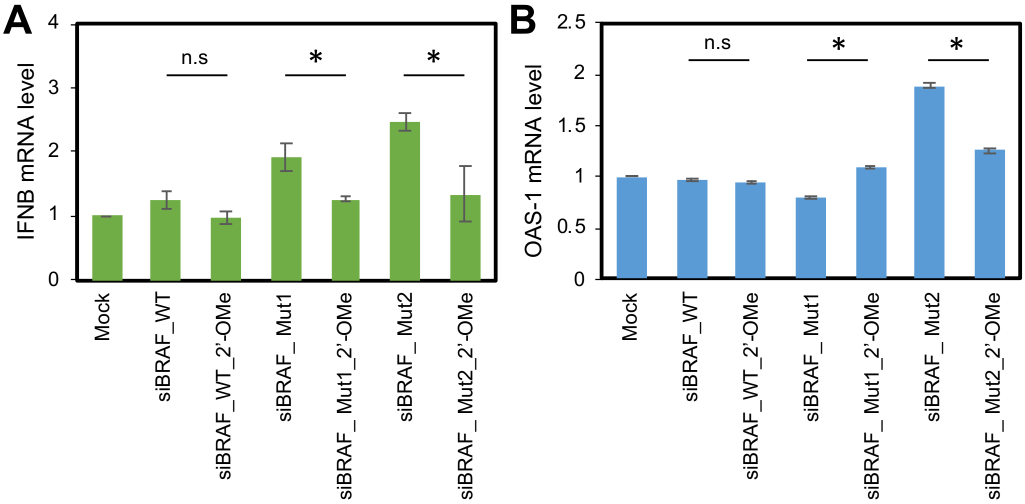

Figure S5. Detection of innate immune response by siRNA. (A) IFN-β and (B) OAS-1 mRNA levels quantified by qRT-PCR using total RNA from BxPC-3 cells transfected according to the same procedure shown in Figure 2B. Their mRNA levels were normalized to that in mock-transfected control cells. The *p*-values for comparisons between each unmodified and 2′-OMe-modified siRNA were determined using Student's *t*-test (*$p < 0.05$). n.s., not significant.

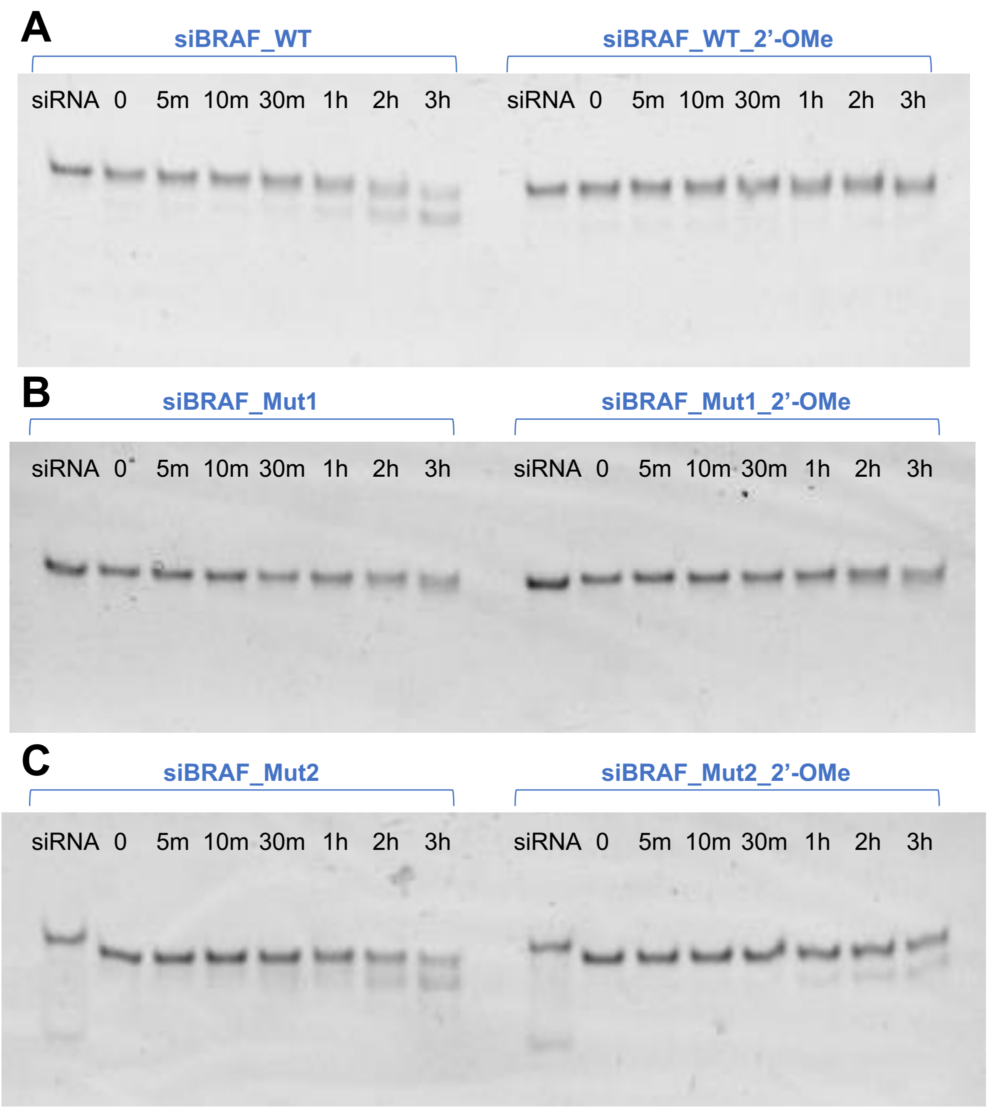

Figure S6. Differences in siRNA stabilities between unmodified and 2′-OMe-modified siRNAs. Comparisons of stabilities between unmodified and 2′-OMe-modified siRNAs in fetal bovine serum: (A) siBRAF WT and siBRAF_WT_2′-OMe, (B) siBRAF_Mut1 and siBRAF_ Mut1_2′-OMe, and (C) siBRAF_Mut2 and siBRAF_ Mut2_2′-OMe. Each siRNA was treated with serum for indicated time, and frozen immediately before electrophoresis. Each "siRNA" lane indicates the full-length band without serum.

Supplementary Tables:

Table S1. SiRNA sequences used for reporter assays, qRT-PCRs, and western blots.

siRNA	Passenger strand (5'→3')	Guide strand (5'→3')
siControl (siGY441)	GCCACAACGUCUAUAUCAUGG	AUGAUAUAGACGUUGUGGCUG
siKRAS	CAGCUAAUUCAGAAUCAUUUU	AAUGAUUCUGAAUUAGCUGUA
siBRAF_Mut&WT	GUUACAAGCCUUCAAAAAUGA	AUUUUUGAAGGCUUGUAACUG
siBRAF_WT	GAAUGUGACAGCACCUACACC	UGUAGGUGCUGUCACAUUCAA
siBRAF_Mut1	GGAAAAUGUUGAAUGCUCAGC	UGAGCAUUCAACAUUUUCCCU
siBRAF_Mut2	GUUGAAUGCUCAGCAGUUACA	UAACUGCUGAGCAUUCAACAU

Table S2. Oligonucleotides sequences inserted into psiCHECK-1 for luciferase reporter assays.

Oligonucleotide name	Sequence (5'→3')
siBRAF_WT&Mut-SM-s	tcgagGTCAATGTTCGGATCAAAAATGAGTCAATGTTCGGATCAAAAATGAGTCAATGTTCGGATCAAAAATGAg
siBRAF_WT&Mut-SM-as	aattcTCATTTTTGATCCGAACATTGACTCATTTTTGATCCGAACATTGACTCATTTTTGATCCGAACATTGACc
siBRAF_WT-SM-s	tcgagAACTTACACTGTCCACCTACACCAACTTACACTGTCCACCTACACCAACTTACACTGTCCACCTACACCg
siBRAF_WT-SM-as	aattcGGTGTAGGTGGACAGTGTAAGTTGGTGTAGGTGGACAGTGTAAGTTGGTGTAGGTGGACAGTGTAAGTTc
siBRAF_Mut1-SM-s	tcgagTCCCTTTTACAACAATGCTCAGCTCCCTTTTACAACAATGCTCAGCTCCCTTTTACAACAATGCTCAGCg
siBRAF_Mut1-SM-as	aattcGCTGAGCATTGTTGTAAAAGGGAGCTGAGCATTGTTGTAAAAGGGAGCTGAGCATTGTTGTAAAAGGGAc
siBRAF_Mut2-SM-s	tcgagTACAACTTACGAGAGCAGTTACATACAACTTACGAGAGCAGTTACATACAACTTACGAGAGCAGTTACAg
siBRAF_Mut2-SM-as	aattcTGTAACTGCTCTCGTAAGTTGTATGTAACTGCTCTCGTAAGTTGTATGTAACTGCTCTCGTAAGTTGTAc
siBRAF_WT&Mut-CM-s	tcgagTACACCTCAGCAGTTACAAGCCTTCAAAAATGAAGTAGGAGg
siBRAF_WT&Mut-CM-as	aattcCTCCTACTTCATTTTTGAAGGCTTGTAACTGCTGAGGTGTAc
siBRAF_WT-CM-s	tcgagAGTGAAAATGTTGAATGTGACAGCACCTACACCTCAGCAGTg
siBRAF_WT-CM-as	aattcACTGCTGAGGTGTAGGTGCTGTCACATTCAACATTTTCACTc
siBRAF_Mut1-CM-s	tcgagGTGATGTGGCAGTGAAAATGTTGAATGCTCAGCAGTTACAAg
siBRAF_Mut1-CM-as	aattcTTGTAACTGCTGAGCATTCAACATTTTCACTGCCACATCACc
siBRAF_Mut2-CM-s	tcgagGGCAGTGAAAATGTTGAATGCTCAGCAGTTACAAGCCTTCAg
siBRAF_Mut2-CM-as	aattcTGAAGGCTTGTAACTGCTGAGCATTCAACATTTTCACTGCCc

s=sense strand, as=antisense strand

CM=complete match, SM=seed match

Lower case is the sequence of restriction enzyme site

Table S3. Primer sequences used for qRT-PCRs.

Primer name	Sequence (5'→3')
BRAF-F	GTCTACAAGGGAAAGTGGCATG
BRAF-R	AGTTGTGGCTTTGTGGAATAGC
Dis-BRAF-WT	AGGCTTGTAACTGCTGAAG
Dis-BRAF-Mut	AGGCTTGTAACTGCTGAAC
KRAS-F	GAGGCCTGCTGAAAATGACTG
KRAS-R	ATTACTACTTGCTTCCTGTAGG
GAPDH-F	TGCACCACCAACTGCTTAG
GAPDH-R	AGAGGCAGGGATGATGTTC
LYRM7-F	ATGCCAGAGCATTAGAAGCAGC
LYRM7-R	ACCAGTTTCAGTGTATTGTGGTCTG
MRPS16-F	TTGCCCTCAACCTAGACAGG
MRPS16-R	CGTTTCCTTCGCAGTCTCTC
RNGTT-F	ACACAGGAACCATTTAGCGTCA
RNGTT-R	CATCGACCAGGTTTGTATTTTCCAG
SSR1-F	ATTGATGGCACAAATCAGCA
SSR1-R	CGCATTAAACAGCAGCAAAA
MAPK8IP3-F	TGGAATGGGCAAAGAAGTGGG
MAPK8IP3-R	TGGCCTCGGACTTCACTCTTTT
SETD1A-F	GATGCGGCCAATCTCCAAGC
SETD1A-R	ATCCATCTTTGCTCATTTACACTGC
SURF4-F	GTCAAGGTTGGTTGGCTGAT
SURF4-R	GCCAGGAGAAACAGGAACAC
TRUB1-F	TGTGTGCAGATGCAGAATGA
TRUB1-R	CCAACTGCAAGGCCATTTAT
GPD2-F	CGGACAACGAGAAGTCGTCAGG
GPD2-R	CATAGGCCAGGTTCATTTGTTTCC
KNSTRN-F	GGGCCTTGATCCAGCTTTAGG
KNSTRN-R	GGTGCAATATCCCATGATTCATCCA
PRKCI-F	ATGGCCACACTTTCCAAGCC
PRKCI-R	GCATCACTGGTTCCTGTGGCA
SAP30-F	CGAGCTGGATAAGAGCGCAAG
SAP30-R	ACCTCCATCATCATCACTCCCTT
PRDM16-F	GAGCCGATGCCGACTTTTGG
PRDM16-R	GCATCATTGCATATGCCTGGTTCT
VAPA-F	TTCAGGAAATGCCAAGAGGT
VAPA-R	TCAACAACTGCCTCACAAGG
VIM-F	CAGGACTCGGTGGACTTCTC
VIM-R	GTCGATGTAGTTGGCGAAGC
GMFB-F	CCTGAACGACAACCTCGCTTC
GMFB-R	CCTTGGTTAGTTCAGCTGTCTGG

RAB2B-F	ACGCGACACCATGACTTATGC
RAB2B-R	AGCTCCAAACTCCACACCTATTG
TMEM65-F	ATCTAGCTGGACTTGGACTTGC
TMEM65-R	AGCCAATAGTCACCCCAACAGC
IFNB-F	AAACTCATGAGCAGTCTGCA
IFNB-R	AGGAGATCTTCAGTTTCGGAGG
OAS1-F	TTGATGCCCTGGGTCAGTTG
OAS1-R	AGAACTCGCCCTCTTTCTGC

F=forward primer; R=reverse primer

Dis= Distinguishable

WT=Wild-type Mut=Mutated
