# Supplementary figures and images for: Knockdown of 15-bp Deletion-Type v-raf Murine Sarcoma Viral Oncogene Homolog B1 mRNA in Pancreatic Ductal Adenocarcinoma Cells Repressed Cell Growth In Vitro and Tumor Volume In Vivo
Source: Cancers (Basel). 2022 Jun 28;14(13):3162. doi: 10.3390/cancers14133162 (PMC9264874; doi:10.3390/cancers14133162)

Figure 1C

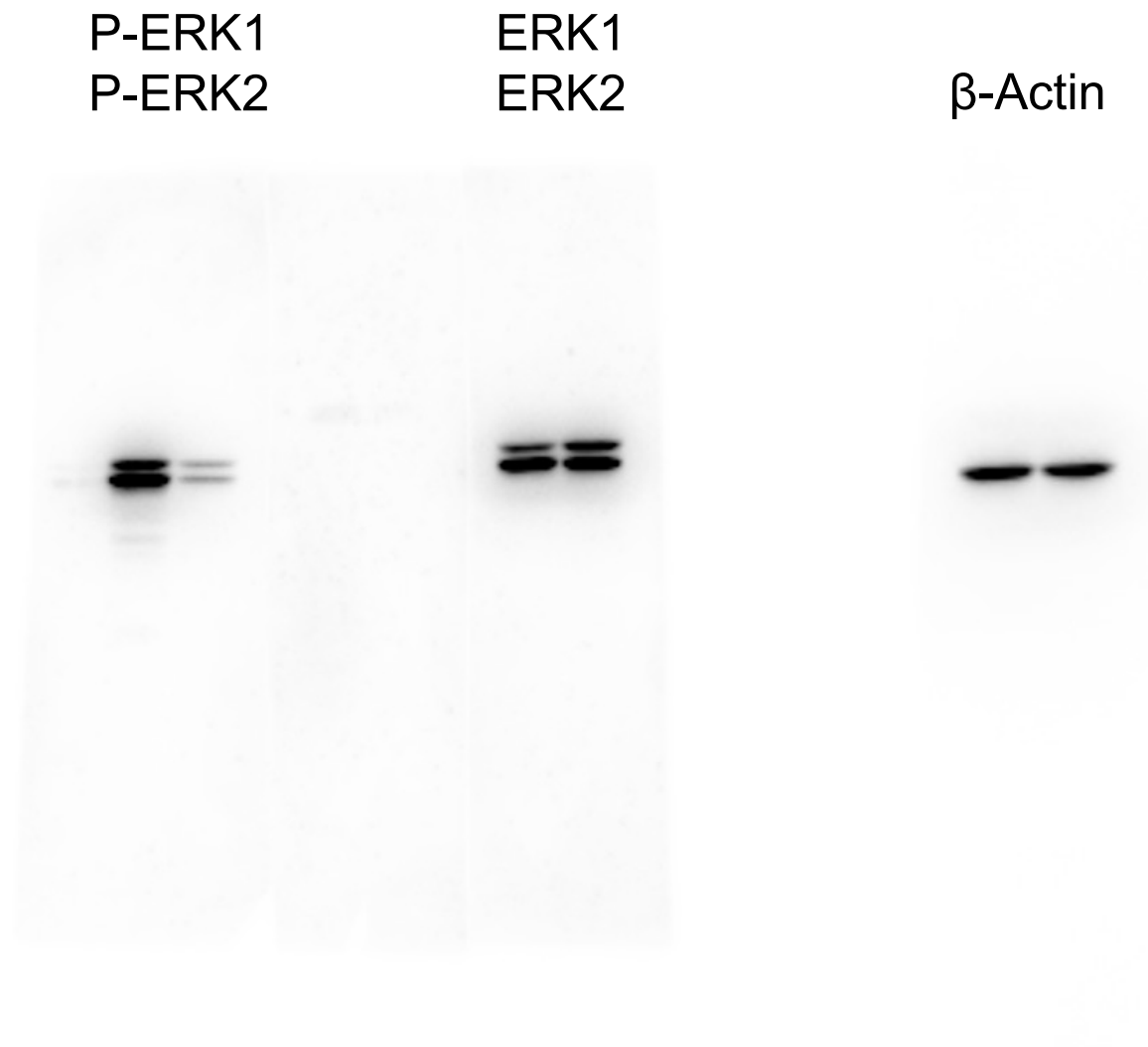

Figure 1F

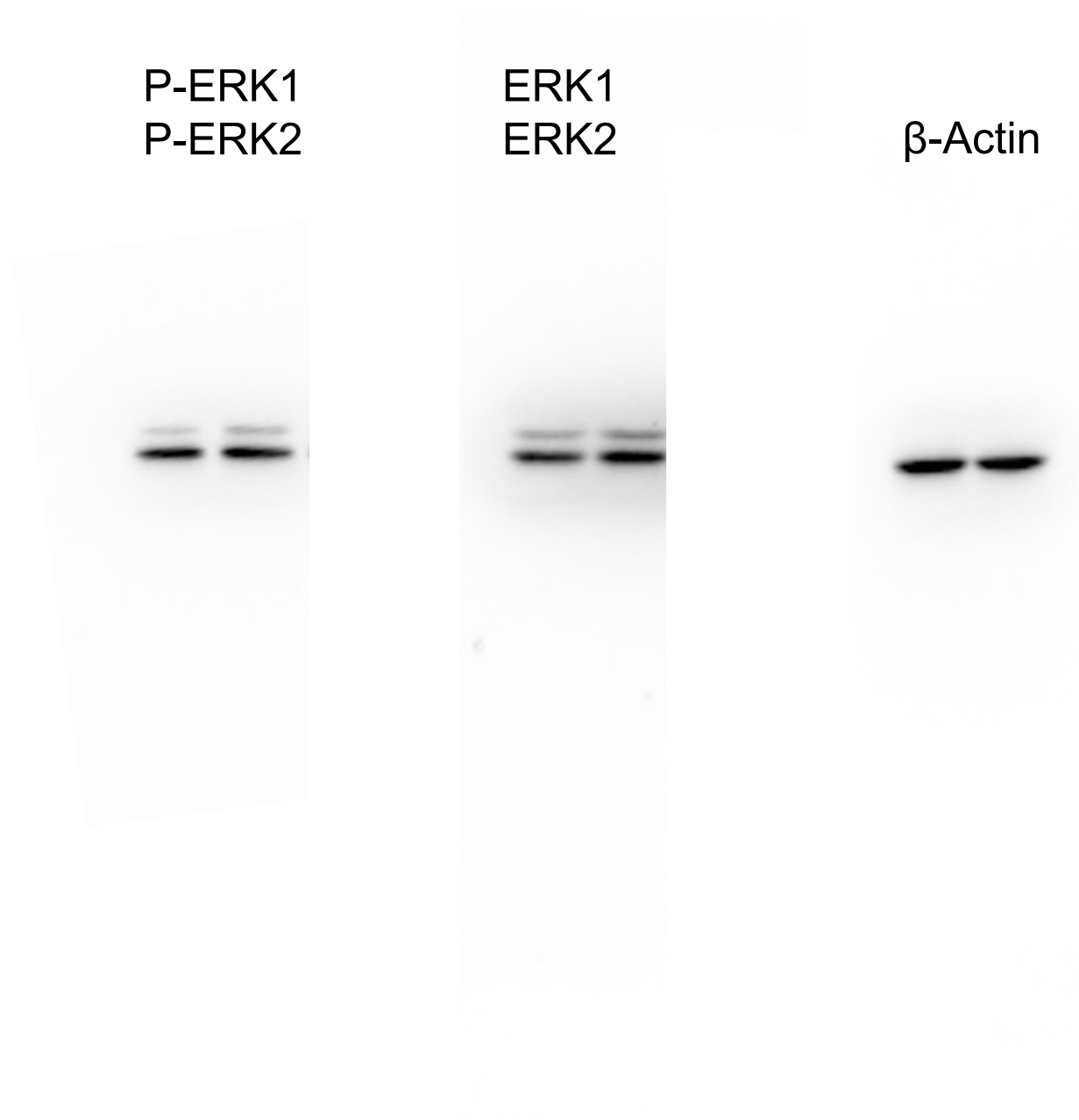

Figure 2F

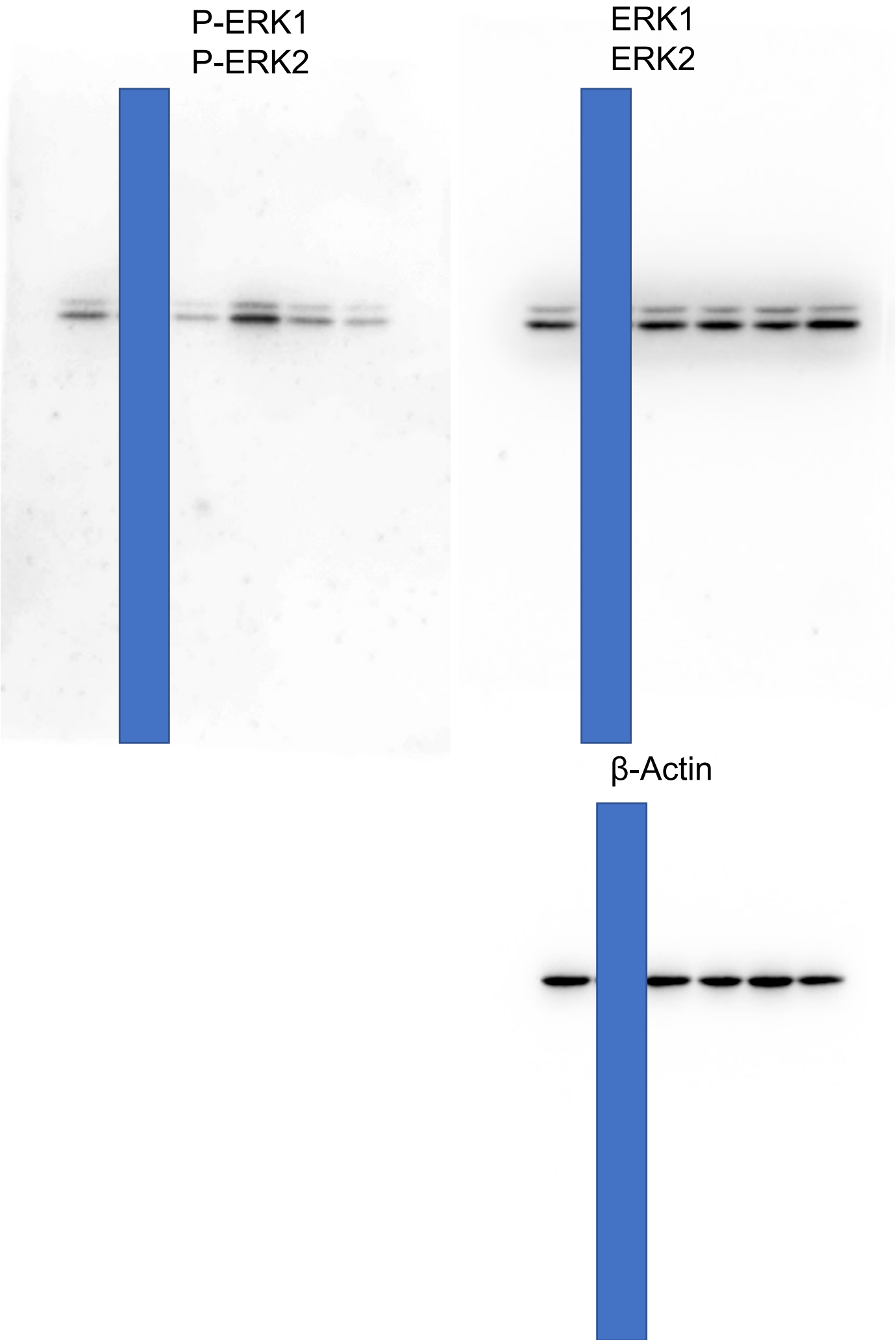

Supplement: Supplementary file 1 [file cancers-14-03162-s001.zip › cancers-1731130-original wester blots figures.pdf]
